# Fatal Case of *Burkholderia gladioli* Pneumonia in a Patient With COVID-19

**DOI:** 10.31486/toj.22.0002

**Published:** 2022

**Authors:** Sanu Rajendraprasad, Zachary A. Creech, Gia Thinh D. Truong, Toan Nguyen, Mounika Addula, Neil Mendoza, Manasa Velagapudi

**Affiliations:** ^1^ Department of Infectious Diseases, Creighton University School of Medicine, Omaha, NE; ^2^ Department of Internal Medicine, Creighton University School of Medicine, Omaha, NE; ^3^ Department of Medicine, Houston Methodist Hospital, Houston, TX

**Keywords:** Burkholderia gladioli, *COVID-19*, *cystic fibrosis*, *pneumonia*

## Abstract

**Background:** *Burkholderia gladioli (B gladioli)* is a rare, gram-negative rod that was initially regarded as a plant pathogen. However, *B gladioli* has been reported as the primary pathogen causing pneumonia in organ transplant recipients and in patients with cystic fibrosis. We report a case of bacterial pneumonia caused by *B gladioli* in a patient hospitalized for coronavirus disease 2019 (COVID-19).

**Case Report:** A 68-year-old male was admitted for acute hypoxic respiratory failure secondary to COVID-19 pneumonia. He was treated with dexamethasone and convalescent plasma, resulting in improvement in the hypoxemia. However, during the latter part of his inpatient stay, the patient developed pneumonia caused by *B gladioli*. The isolate of *B gladioli* was sensitive to meropenem, levofloxacin, and trimethoprim/sulfamethoxazole and intermediate to ceftazidime. He was treated with meropenem and levofloxacin. Despite treatment, the patient developed acute respiratory distress syndrome with multiorgan failure, suffered cardiac arrest, and died.

**Conclusion:** To the best of our knowledge, this case is the first report of *B gladioli* coinfection in a patient hospitalized for COVID-19 and provides insight into the possible detrimental outcome of *B gladioli* and COVID-19 coinfection.

## INTRODUCTION

*Burkholderia gladioli* (*B gladioli*) is a rare, aerobic, gram-negative rod that was initially regarded as an environmental microbe and plant pathogen.^1^ However, reports have identified *B gladioli* as the causative agent of pneumonia in organ transplant recipients and patients with cystic fibrosis,^2,3^ and *B gladioli* has been shown to be an opportunistic pathogen in patients with chronic granulomatous disease or immunosuppression.^4^ Clinically, *B gladioli* can present with upper respiratory infection symptoms, septicemia, pulmonary abscesses, osteomyelitis, corneal inflammation, and even lymphadenitis.^4^ While the pathogenesis of *B gladioli* is relatively unclear, laboratory investigations have indicated that strains of the bacteria are sensitive to the complement-mediated lysis of human serum, giving immunocompetent individuals a natural defense against the organism.^5^

Recent (2020, 2021) studies have shown that coronavirus disease 2019 (COVID-19) infection can increase susceptibility to opportunistic infections or to reactivations of prior infections such as tuberculosis and mycobacteria.^6,7^ The consequences and implications of infection with COVID-19 are still being studied, and to our knowledge, this report is the first case of coinfection of *B gladioli* and severe acute respiratory syndrome coronavirus 2 (SARS-CoV-2), the virus that causes COVID-19. This case suggests that the lung damage caused by COVID-19 could be a predisposing factor for the development of rare *B gladioli* infection.

## CASE REPORT

A 68-year-old male presented to the emergency department (ED) with fatigue, shortness of breath, and poor appetite for the prior 10 days. The patient had a medical history of hypertension, diabetes mellitus type 2, chronic kidney disease stage 3, and complete heart block requiring a pacemaker. On arrival in the ED, he was afebrile and had an oxygen saturation of 45% on room air. He was placed on high-flow oxygen at 15 L/min. Chest x-ray showed bilateral interstitial infiltrates. The patient's condition deteriorated; his increasing oxygen requirements necessitated intubation and ventilation 2 days after his initial admission. Initial workup showed a white blood cell count of 6.0 K/μL (reference range, 4.0-12.0 K/μL), lactate dehydrogenase of 745 U/L (reference range, 84-246 U/L), D-dimer of 5.71 mg/L FEU (reference, <0.50 mg/L FEU), ferritin of 5,934 ng/mL (reference range, 22-388 ng/mL), fibrinogen of 585 mg/dL (reference range, 200-400 mg/dL), and procalcitonin of 7.62 ng/mL (reference, <0.05 ng/mL). Given the high index of clinical suspicion for COVID-19, the patient was admitted to the intensive care unit (ICU) for management of acute hypoxic respiratory failure. COVID-19 polymerase chain reaction test on nasopharyngeal swab detected SARS-CoV-2. The patient was started on 2 g of intravenous (IV) ceftriaxone every 24 hours and 500 mg of azithromycin daily for empiric treatment of community-acquired pneumonia. However, azithromycin was discontinued after urine *Legionella* testing was negative. The patient continued ceftriaxone for 7 days. He was also treated with 1 dose of approximately 200 mL of convalescent plasma on day 2 of admission, and dexamethasone starting with 20 mg IV once daily for 5 days, followed by 10 mg IV for an additional 5 days.

The patient's renal function continued to worsen. Given the patient's ongoing hypotension (98/50 mm Hg) and increasing pressor requirement, he was transitioned to continuous renal replacement therapy. With the initiation of prone positioning protocol, the patient's ventilator requirements improved from 100% fraction of inspired oxygen (FiO_2_) to 40% FiO_2_, and his positive end-expiratory pressure decreased to 5 cm H_2_O from a high of 12 cm H_2_O. Additionally, the patient's procalcitonin decreased to 0.66 ng/mL from a peak of 7.62 ng/mL.

However, on day 13 of his hospital stay in the ICU, the patient's health status started to decline. He was febrile with a maximum temperature of 101 °F (38.3 °C) and had increased ventilator requirements with positive end-expiratory pressure of 16 cm H_2_O and FiO_2_ of 90%. Workup showed leukocytosis with a white blood cell count of 24.4 K/μL. Because of the patient's worsening oxygen requirements and to empirically treat ventilator-associated pneumonia, the patient's antimicrobial therapy was expanded: 2 g cefepime IV every 12 hours for 5 days, 2 g vancomycin followed by level-based dosing with a target trough of 15 μg/mL for 5 days, and 1 dose of 750 mg of levofloxacin IV followed by 500 mg every 48 hours for a total of 10 days. The patient's antibiotic dosage was adjusted accordingly for his decreased renal function and creatinine clearance of 18 mL/min/1.73 m^2^ (reference, ≥90 mL/min/1.73 m^2^). Computed tomography angiogram of the chest showed bilateral extensive pulmonary consolidation (Figure).

**Figure.**
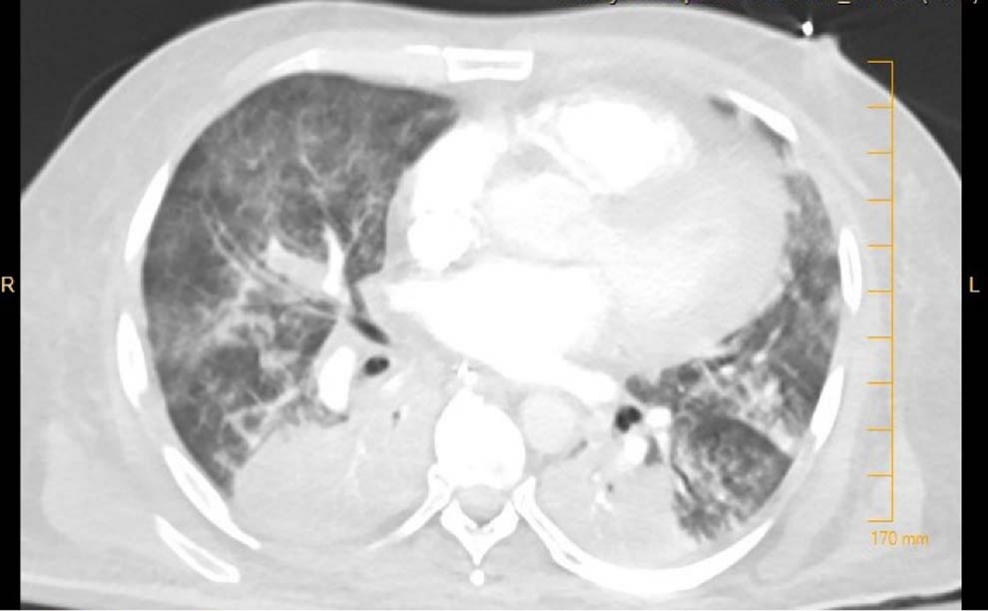
Computed tomography angiogram shows extensive bilateral pulmonary consolidation as a result of ventilator-associated pneumonia coinfection of *Burkholderia gladioli* and coronavirus disease 2019 (COVID-19).

Sputum cultures of tracheal aspirates on day 14 of the patient's hospital stay grew *B gladioli*. *B gladioli* was detected using matrix-assisted laser desorption/ionization time-of-flight (MALDI-TOF) mass spectrometry and confirmed with VITEK MS (bioMérieux). The isolate was intermediate to ceftazidime and susceptible to levofloxacin and trimethoprim/sulfamethoxazole. However, after 7 days of treatment, repeat cultures from tracheal aspirate on day 21 still showed *B gladioli.* Given the patient's lack of improvement on vancomycin, cefepime, and levofloxacin antimicrobial therapy, the Infectious Diseases service was consulted, and cefepime was switched to IV meropenem at a dose of 500 mg every 8 hours for a duration of 6 days. Significant laboratory values of the patient's clinical progression from his day of admission to day 13, day 19, and day 24 of hospitalization are shown in the Table.

**Table.** Significant Laboratory Values During Patient's Hospitalization

Variable	Reference	Day 1	Day 13	Day 19	Day 24
White blood cell count, K/μL	4.0-12.0	6.0	24.4	8.6	23.5
Creatinine, mg/dL	0.60-1.30	5.50	3.39	4.03	1.27
C-reactive protein, mg/mL	≤9.0	177	N/A	191	N/A
Creatinine clearance, mL/min/1.73m^2^	≥90	15	18	15	59
Procalcitonin, ng/mL	<0.05	7.62	0.66	5.86	4.64
Lactate dehydrogenase, U/L	84-246	745	N/A	328	N/A
D-dimer, mg/L FEU	<0.50	5.71	N/A	8.09	N/A

N/A, not available.

The patient remained prone, intubated, sedated, and paralyzed on rocuronium. He required a total of 4 pressors, including phenylephrine, norepinephrine, and vasopressin. Midodrine was also used for augmentation of blood pressure. Despite all therapeutic interventions, the patient continued to deteriorate with progression to acute respiratory distress syndrome with multiorgan failure. He suffered cardiac arrest and died on day 25 of his ICU stay.

## DISCUSSION

SARS-CoV-2 is the primary pathogen responsible for the worldwide pandemic that started in December 2019. The clinical course of COVID-19 infection can range from mild respiratory symptoms to complications, including secondary bacterial pneumonia. A retrospective multicenter study of 476 patients with COVID-19 pneumonia reported that secondary bacterial pneumonia is associated with poor outcomes and death.^8^ *Streptococcus pneumoniae, Klebsiella pneumoniae, Haemophilus influenzae, Escherichia coli, Staphylococcus aureus, Pseudomonas aeruginosa*, and *Aspergillus* have been reported to cause secondary bacterial pneumonias.^9^

To our knowledge, this case is the first-ever reported incidence of *B gladioli* secondary bacterial pneumonia in an immunocompetent patient with no underlying lung disease who acquired COVID-19, although *B cepacia* coinfections with COVID-19 have been reported in an interhospital outbreak and in a patient with cystic fibrosis.^10,11^ *B gladioli* is an aerobic gram-negative bacillus, previously known as *Pseudomonas gladioli* and *P marginata*.^1^ It was first recognized as a plant pathogen of gladiolus and iris flowers.^1^ In 1989, *P gladioli* was initially described in the medical literature as a colonizing organism in patients with cystic fibrosis.^12^

A 6-year retrospective study in 2007 examining the impact of *B gladioli* in 33 patients with cystic fibrosis showed that 40% of patients with cystic fibrosis were chronically infected with *B gladioli* as evidenced by ≥2 positive cultures separated by at least 6 months.^13^ Chronic infection was associated with resistance to more than 2 antibiotic groups on initial culture and failure to eradicate after antibiotic therapy.^13^ These cases highlight the potential role of *B gladioli* as a colonizer in patients with chronic infections. The paucity of literature on *B gladioli* and its reported antibiotic resistance can potentially lead to a complicated clinical outcome, especially in cases of coinfection. Other case reports have demonstrated the role of *B gladioli* as an opportunistic pathogen in patients with chronic granulomatous disease, AIDS, and diabetes mellitus and in liver and lung transplant recipients.^14,15^ Potential complications resulting from *B gladioli* infection reported in the literature are pneumonia, mediastinal abscess, bacteremia, osteomyelitis, maxillary sinusitis, and early neonatal and nosocomial sepsis in newborns.^13,15-18^ No person-to-person transmission has ever been noted.^2^

Our patient's onset of hypoxemia, fever, and elevated procalcitonin and white blood cell count during his ICU stay coincided with the identification of *B gladioli* from tracheal isolates. The patient's change in clinical course was likely the result of a secondary bacterial infection of *B gladioli.* This case occurred during May 2020. While treatment guidelines for COVID-19 were not established at that time, the patient was not a candidate for treatments such as tocilizumab or remdesivir because of his severe renal impairment. The patient was not vaccinated for COVID-19, which could have potentially contributed to his clinical course. *Burkholderia* spp infections often result from outbreaks in hospitals; however, our case was isolated with no other outbreak during this time period.^19^ Given the potential role of *B gladioli* as an opportunistic pathogen, we hypothesize that the underlying structural damage caused by COVID-19 predisposed our patient to *B gladioli* pneumonia.

The treatment of this bacterium can be challenging because of various antibiotic-resistant mechanisms, including the production of beta-lactamases, plasmid-mediated antimicrobial resistance, and biofilm formation.^17^ Additionally, coinfection of *B gladioli* with other pathogens is common, and antimicrobial therapy should be based on susceptibility testing*. B gladioli* appears to be colistin-resistant but susceptible to aminoglycosides and ciprofloxacin, imipenem, and ticarcillin-clavulanic acid, although aminoglycoside-resistant isolates may emerge in the course of chronic infections.^4^ One case report showed that a reduction of immunosuppression combined with antimicrobials led to clearance of *B gladioli* infection but ultimately led to the patient's death from an invasive fungal infection of the lungs.^20^ Infection with this bacterium is often associated with a poor prognosis, as with our patient.^20^

## CONCLUSION

To our knowledge, this case is the first report of coinfection of *B gladioli* with SARS-CoV-2. Despite early and aggressive interventions, the patient died from cardiac arrest. Our case adds to the existing literature of COVID-19 and highlights the role of *B gladioli* as an opportunistic pathogen. Accurate management of nosocomial bacterial infections and prevention of secondary infections may improve patient outcomes.

## Notes

*This article meets the Accreditation Council for Graduate Medical Education and the American Board of Medical Specialties Maintenance of Certification competencies for Patient Care and Medical Knowledge.*
